# Non-Cholesterol Sterol Levels Predict Hyperglycemia and Conversion to Type 2 Diabetes in Finnish Men

**DOI:** 10.1371/journal.pone.0067406

**Published:** 2013-06-28

**Authors:** Henna Cederberg, Helena Gylling, Tatu A. Miettinen, Jussi Paananen, Jagadish Vangipurapu, Jussi Pihlajamäki, Teemu Kuulasmaa, Alena Stančáková, Ulf Smith, Johanna Kuusisto, Markku Laakso

**Affiliations:** 1 Department of Medicine, University of Eastern Finland and Kuopio University Hospital, Kuopio, Finland; 2 Institute of Public Health and Clinical Nutrition, University of Eastern Finland, Kuopio, Finland; 3 Department of Medicine, Division of Internal Medicine, University of Helsinki, Helsinki, Finland; 4 Department of Medicine, University of Eastern Finland, Kuopio, Finland; 5 Department of Molecular and Clinical Medicine, Sahlgrenska Academy at the University of Gothenburg, Gothenburg, Sweden; Cornell University College of Veterinary Medicine, United States of America

## Abstract

We investigated the levels of non-cholesterol sterols as predictors for the development of hyperglycemia (an increase in the glucose area under the curve in an oral glucose tolerance test) and incident type 2 diabetes in a 5-year follow-up study of a population-based cohort of Finnish men (METSIM Study, N = 1,050) having non-cholesterol sterols measured at baseline. Additionally we determined the association of 538,265 single nucleotide polymorphisms (SNP) with non-cholesterol sterol levels in a cross-sectional cohort of non-diabetic offspring of type 2 diabetes (the Kuopio cohort of the EUGENE2 Study, N = 273). We found that in a cross-sectional METSIM Study the levels of sterols indicating cholesterol absorption were reduced as a function of increasing fasting glucose levels, whereas the levels of sterols indicating cholesterol synthesis were increased as a function of increasing 2-hour glucose levels. A cholesterol synthesis marker desmosterol significantly predicted an increase, and two absorption markers (campesterol and avenasterol) a decrease in the risk of hyperglycemia and incident type 2 diabetes in a 5-year follow-up of the METSIM cohort, mainly attributable to insulin sensitivity. A SNP of *ABCG8* was associated with fasting plasma glucose levels in a cross-sectional study but did not predict hyperglycemia or incident type 2 diabetes. In conclusion, the levels of some, but not all non-cholesterol sterols are markers of the worsening of hyperglycemia and type 2 diabetes.

## Introduction

Type 2 diabetes is associated with atherogenic dyslipidemia [1] increasing the risk of cardiovascular mortality and morbidity [2]. Abnormalities in cholesterol metabolism in type 2 diabetes include enhanced cholesterol synthesis and reduced cholesterol absorption [3]–[6]. Also insulin resistance and obesity have been previously associated with elevated cholesterol synthesis and low cholesterol absorption in cross-sectional studies [7]–[10]. However, the mechanisms underlying the association of non-cholesterol sterol levels with hyperglycemia have remained unclear. Atherogenic dyslipidemia predicts the development of type 2 diabetes [1], and therefore sterols associated with cholesterol synthesis and absorption could also contribute to the risk of the development of hyperglycemia and type 2 diabetes.

Single nucleotide polymorphisms (SNPs) in the sitosterolemia genes *ABCG5* and *ABCG8*, encoding the ATP-binding cassette sub-family G member proteins, have been shown to regulate cholesterol absorption [11]–[13]. These transporters actively efflux plant sterols and, to a lesser extent cholesterol, back into the intestinal lumen. In a previous genome-wide association study SNPs rs4245791 and rs41360247 in/near *ABCG8* were associated with serum phytosterol levels [14]. The impact of SNPs in *ABCG5/8*, and possible other, yet undiscovered SNPs on the association between non-cholesterol sterol levels and glucose metabolism is largely unknown.

To investigate in more detail the association of non-cholesterol sterols with the risk of the worsening of hyperglycemia and incident type 2 diabetes we addressed 1) the relationship of cholesterol synthesis markers (squalene, cholestenol, lathosterol, and desmosterol) and absorption markers (campesterol, sitosterol, avenasterol, and cholestanol) with fasting and 2-hour glucose levels in a cross-sectional setting, 2) the association of non-cholesterol sterol levels as predictors for the worsening of hyperglycemia and the development of incident type 2 diabetes in a 5-year follow-up of the METSIM (METabolic Syndrome In Men) Study, and 3) the role of insulin sensitivity, insulin secretion and genetic factors in this relationship.

## Subjects and Methods

### Participants and Clinical Measurements

#### METSIM study

Non-cholesterol sterol levels were determined in a random sample of 1,050 consequently examined men from the population-based METSIM Study including 10,197 men. This study has been described in detail elsewhere [15]. Glucose tolerance was evaluated according to the American Diabetes Association criteria [16]. Participants with previously diagnosed diabetes mellitus or receiving statin or ezetimibe medication were excluded, leaving 746 men for statistical analysis. A total of 474 METSIM participants (64%) have been re-examined during an ongoing follow-up study [follow-up time 5.4±0.3 years, mean±SD); 25.1% with normal glucose tolerance, 51.3% with isolated impaired fasting (IFG) glucose, 3.6% with isolated impaired glucose tolerance (IGT), 12.1% with IFG and IGT, and 7.9% with incident type 2 diabetes]. Body mass index (BMI) was calculated as weight (kg) divided by the height (m) squared. *Ethical Approval* The study was approved by the Ethics Committee of the University of Kuopio and Kuopio University Hospital, conducted in accordance with the principles of the Declaration of Helsinki, and all study participants gave written informed consent.

#### Kuopio cohort of the EUGENE2 study

The participants were healthy, non-diabetic offspring of patients with type 2 diabetes, as previously described in detail [17]. The probands were randomly selected among type 2 diabetic patients living in the region of Kuopio, Finland. One of the parents had to have type 2 diabetes and the other parent normal glucose tolerance in an OGTT. Altogether 273 offspring (128 males, 145 females) were included in the current study aiming to identify SNPs regulating sterol levels. *Ethical Approval* The study was approved by the Ethics Committee of the University of Kuopio and Kuopio University Hospital, conducted in accordance with the principles of the Declaration of Helsinki, and all study participants gave written informed consent.

### Sterol and Lipid Measurements

The methods to measure sterol and lipid levels have been described in detail elsewhere [7]. Briefly, **s**erum cholesterol, cholesterol precursors (squalene, cholestenol, lathosterol, and desmosterol), campesterol, sitosterol and avenasterol (plant sterols), and cholestanol, a metabolite of cholesterol, were quantified from non-saponifiable serum material by capillary gas chromatography (Agilent 6890N Network GC System, Agilent Technologies, Wilmington, DE) equipped with a 50 m long Ultra 2 capillary column (5% Phenyl-methyl siloxane) (Agilent Technologies, Wilmington, DE) [18]. α-cholestane was used as the internal standard. The inter-assay coefficients of variation for sterols at relevant concentrations were as follows: cholesterol 3.2%, cholestanol 2.7%, desmosterol 6.0%, lathosterol 3.7%, campesterol 1.8%, sitosterol 2.4%, and avenasterol 4.1%, respectively. External quality control samples were run and analyzed with every 25 study samples. The serum values were expressed as 10^2^ µmol/mmol of cholesterol (called ratio in the text) by dividing non-cholesterol sterol concentrations with the cholesterol value of the same GC run in order to eliminate the changing concentrations of sterol transporters, mainly LDL. The ratios to cholesterol of serum cholesterol precursors reflect whole-body cholesterol synthesis and those of plant sterols and cholestanol cholesterol absorption.

### Insulin Sensitivity and Insulin Secretion Indices

Matsuda index of insulin sensitivity (ISI) was calculated as previously reported [15]. An index of early-phase insulin secretion during an OGTT, InsAUC_0–30_/GluAUC_0–30_, was calculated as (fasting insulin +30 min insulin)/(fasting glucose +30 min glucose) (pmol/mmol) [19].

### Genetic Analyses

#### Genotyping

Genotyping for the participants of the EUGENE2 Study was carried out at the Finnish Genome Center in Helsinki, Finland. Genotyping was performed with the commercial release of the Infinium HumanHap 550 k version 3 SNP microarrays (Illumina, San Diego, CA). Briefly, 750 ng of DNA was used in genotyping according to the manufacturer’s protocol (Illumina). Genotyping for the METSIM Study participants was performed using the TaqMan Allelic Discrimination Assays (Applied Biosystems) or the Sequenom MassARRAY platform at the University of Eastern Finland.

#### Genotyping quality assessment and control in the EUGENE2 study

Software package PLINK v1.07 [20] was used for quality assessment and control for the genotyping data. Gender calls from X chromosome genotype data was verified to be in concordance with the reported gender of each individual. To verify known familial relationships and to detect not reported first-degree cryptic relationships, pairwise identity-by-descent analysis was performed. Individuals with genotyping call rates <99% were excluded. All of the 273 samples passed this criterion. Total genotyping call rate in remaining individuals was ≥99.2%. Genotypes advanced to the actual association analysis if they passed the following quality control criteria 1) had a >95% genotype call rate (4,201 markers failed), 2) had a minor allelic frequency (MAF) >0.1% (18,720 markers failed) and 3) demonstrated Hardy-Weinberg Equilibrium (HWE) with a *P*>1×10^−5^ (575 markers failed). In addition, 317 heterozygous haploid genotypes were set to be missing. Out of the 561,466 genotypes, a total of 538,265 markers passed these quality control criteria. For each genotype, summary information about MAF, HWE and call rates were recorded, and this information was used when evaluating marker quality for replication.

#### Genotype replication

Most significant SNPs from the genome-wide association analysis were replicated in the METSIM cohort. SNPs were selected for replication based on statistical significance, effect size, linkage disequilibrium structure and allele frequencies. Priority was given to genotypes that showed strong associations with several sterol measurements. Total of 177 SNPs were genotyped. Genotypes advanced to the association analysis if they passed the following quality control criteria 1) had a >99% genotype call rate (20 markers failed), 2) had a MAF >5% (30 markers failed), and 3) demonstrated HWE with a *P*>0.001 (13 markers failed). Out of the 177 genotypes, a total of 134 markers passed these quality control criteria.

#### Genome-wide association analysis

Software package GenABEL [21] was used for genome-wide association analysis of the EUGENE2 data. Sterol measurements were transformed to log10-scale to adjust for skewed distributions. To account for the family structures in the data, Identity-by-state kinship matrix between the subjects was calculated and then used within mixed model score test for association in related people [22]. Gender specific differences in the levels of non-cholesterol levels have been previously reported [23] and higher levels of cholesterol synthesis markers squalene, lathosterol and desmosterol were also observed in men as compared women in the EUGENE2 Kuopio cohort (data not shown). Therefore, gender, together with age and BMI were included in the analysis as covariates and the additive genetic model was applied.

#### Replication analysis

PLINK was used to analyze the data from the METSIM replication study. Sterol measurements were transformed to log10-scale to adjust for skewed distributions. Linear regression including age and BMI as a covariate and assuming an additive genetic model was used.

#### Meta-analysis

Joint fixed-effects meta-analysis combining results from both Stage 1 (genome-wide association analysis) and Stage 2 (replication study) was performed using PLINK. Minor alleles from Stage 1 analysis were used as reference alleles.

#### Replication of ABCG8 rs4299376 in the entire METSIM cohort

Genotyping of rs4299376 of *ABCG8* in the entire METSIM cohort was performed with TaqMan Allelic Discrimination Assay (Allied Biosystems). Call rate was 100% and the error rate 0%.

### Statistical Analysis

Statistical analyses were conducted using SPSS version 19 (SPSS, Chicago, IL). All sterol to cholesterol ratios, glucose, BMI, insulin sensitivity index (Matsuda ISI) and insulin secretion index (InsAUC_0–30_/GluAUC_0–30_) were log-transformed to correct for their skewed distribution. Sterols were compared across the fasting and 2-hour glucose categories using the general linear model. *P*≤0.006 (corrected for 8 tests by Bonferroni method) was considered statistically significant. Correlations between sterol levels and indices of insulin sensitivity and insulin secretion were evaluated with partial correlations. Associations between baseline sterol levels and glucose area under the curve (AUC) at follow-up were evaluated using linear regression analysis. Associations between baseline sterol levels and incident type 2 diabetes were evaluated with Cox regression analysis (participants having incident type 2 diabetes at follow-up *vs*. participants having NGT/NFG and HbA1c <6.5% at baseline and follow-up examinations, N = 97). Incident type 2 diabetes at follow-up was defined by the American Diabetes Association criteria [24] as follows: fasting plasma glucose (FPG) ≥7.0 mmol/l or 2-h plasma glucose (2hPG) ≥11.1 mmol/l or HbA1c ≥6.5%, or by a physician-based diagnosis between baseline and follow-up examinations and the use of anti-diabetic medication. For statistical analyses of non-cholesterol sterols as predictors for hyperglycemia or incident type 2 diabetes, *P* value adjusted for multiple comparisons (*P*≤0.006 corrected for 8 traits) by Bonferroni method for considered as statistically significant. For GWAS analysis, *P* value adjusted for multiple comparisons (*P*<6×10^−9^ corrected for 8 traits) by Bonferroni method was considered to be statistically significant. For genetic association analysis, unstandardized effect sizes [B (SE)] per copy of the risk allele were estimated by linear regression analysis adjusted for age, BMI, smoking and physical activity, using untransformed dependent variables. Variables not normally distributed were log-transformed for statistical analyses.

## Results

### Associations of Sterols with Glucose Levels, Insulin Sensitivity and Insulin Secretion

After the exclusion of participants with previously diagnosed diabetes mellitus and participants on statin medication, a total of 746 men were included in statistical analyses. Their mean age was 57.6 years (SD 5.8), BMI 27.1 kg/m^2^ (SD 3.9), 18.8% of them were current smokers, and 63.8% were physically active (undertaking regular physical exercise at least once a week for ≥30 min). Mean FPG at baseline was 5.6 mmol/l (SD 0.6) and 2hPG 6.1 mmol/l (SD 2.20). Levels of sterols at baseline are given in Table S1.

**Figure 1:**
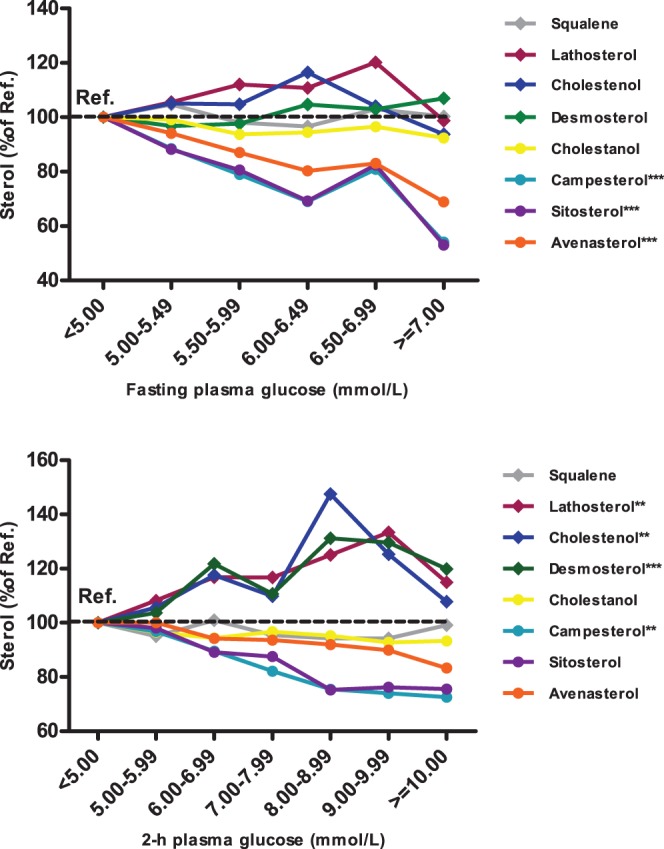
Non-cholesterol sterols indicating cholesterol synthesis (diamonds) and absorption (circles) in relation to hyperglycemia, according to the categories of fasting (FPG) and 2-hour plasma glucose (2hPG) levels (N = 746). Data points represent unadjusted means of sterol levels in each glucose category. *P* values were calculated using general linear model adjusted for age, BMI, smoking, and physical activity: **P<0.01, ***P<10^−5^. Individuals with previously diagnosed diabetes and on statin or ezetimibe treatment were excluded.

As shown in Figure 1 the levels of three absorption markers, campesterol, sitosterol and avenasterol were significantly reduced (*P*<1×10^5^) as a function of increasing FPG levels, but no significant changes were observed for cholesterol synthesis markers. Desmosterol significantly (*P*<1×10^−5^) and lathosterol and cholestenol nominally (*P*<0.01) increased as a function of increasing 2hPG levels, whereas the levels of absorption markers decreased (campesterol, *P*<0.01).

The associations of sterol levels with insulin secretion and insulin sensitivity are shown in Table 1. Synthesis markers, of which desmosterol particularly, correlated inversely with Matsuda ISI, and positively with insulin secretion. In contrast, all markers of cholesterol absorption correlated positively with Matsuda ISI, and cholestanol correlated inversely with insulin secretion. Partial correlations of synthesis markers and cholestanol with insulin secretion lost their statistical significance after the adjustment for Matsuda, suggesting that low insulin sensitivity is the primary mechanism explaining the association of sterols indicating cholesterol synthesis with glucose levels. Similarly, the correlations of the three absorption markers, campesterol, sitosterol and avenasterol were also more significant with insulin sensitivity than with insulin secretion.

**Table 1:** Partial correlations of sterol levels indicating cholesterol synthesis and cholesterol absorption with insulin sensitivity and insulin secretion adjusted for age, BMI, smoking and physical activity.

Sterol, adjusted for total cholesterol	Matsuda ISI		InsAUC_0–30_/GluAUC_0–30_		InsAUC_0–30_/GluAUC_0–30_ adjusted for Matsuda ISI	
	r	*P*	r	*P*	r	*P*
***Synthesis markers***						
Squalene	−0.122	**0.001**	0.119	**0.001**	0.056	0.133
Lathosterol	−0.230	**2.9E-10**	0.175	**1.9E-06**	0.044	0.232
Cholestenol	−0.191	**1.9E-07**	0.147	**6.6E-05**	0.040	0.278
Desmosterol	−0.362	**4.6E-24**	0.228	**4.6E-10**	0.009	0.807
***Absorption markers***						
Cholestanol	0.229	**4.0E-10**	−0.189	**2.8E-07**	−0.064	0.084
Campesterol	0.252	**4.9E-12**	−0.069	0.063	0.113	**0.002**
Sitosterol	0.222	**1.2E-09**	−0.067	0.070	0.091	0.014
Avenasterol	0.137	**2.0E-04**	−0.006	0.878	0.103	**0.006**

Individuals with previously diagnosed diabetes or on statin or ezetimibe treatment were excluded (N = 728–730). Bold types indicate statistical significance with *P*≤0.006.

The correlations of sterol levels with FPG and 2hPG were considerably weaker than those with insulin sensitivity as shown in Table 2. Synthesis markers did not correlate significantly with FPG levels but campesterol, sitosterol and avenasterol had significant inverse correlations with FPG. These correlations considerably weakened after the adjustment for both insulin sensitivity and insulin secretion, whereas no major changes were observed when adjusted for insulin secretion only. Results for 2hPG were quite similar with the exception of higher correlations of synthesis markers with 2hPG than with FPG. Adjustment for all confounding factors and additionally for insulin sensitivity and insulin secretion abolished all statistically significant associations. Taken together, these results provide evidence that the association of glucose metabolism with sterols is mainly mediated by low insulin sensitivity.

**Table 2:** Partial correlations between sterol levels and fasting plasma glucose (FPG), and 2-hour plasma glucose (2hPG), adjusted for age, BMI, smoking, physical activity, and additionally for insulin sensitivity and insulin secretion.

A)												
Sterol, adjusted for total cholesterol	FPG			FPG, adjusted for Matsuda ISI			FPG, adjusted for InsAUC_0–30_/GluAUC_0–30_		FPG, adjusted for InsAUC_0–30_/GluAUC_0–30_ and Matsuda ISI			
	r	*P*		r	*P*		r	*P*	r		*P*	
***Synthesis markers***												
Squalene	−0.029	0.427		−0.091	0.013		−0.010	0.779	−0.073		0.050	
Lathosterol	0.008	0.836		−0.085	0.022		0.055	0.141	−0.073		0.049	
Cholestenol	−0.012	0.736		−0.094	0.011		0.022	0.549	−0.089		0.017	
Desmosterol	0.072	0.051		−0.088	0.017		0.121	**0.001**	−0.106		**0.004**	
***Absorption markers***												
Cholestanol	−0.072	0.051		0.018	0.627		−0.119	**0.001**	−0.028		0.444	
Campesterol	−0.203	**2.3E-08**		−0.126	**0.001**		−0.232	**2.2E-10**	−0.071		0.055	
Sitosterol	−0.183	**5.4E-07**		−0.117	**0.002**		−0.212	**7.2E-09**	−0.077		0.038	
Avenasterol	−0.190	**1.7E-07**		−0.160	**1.4E-05**		−0.206	**2.0E-08**	−0.123		**0.001**	
**Sterol, adjusted for total cholesterol**	**2hPG**			**2hPG, adjusted for Matsuda ISI**			**2hPG, adjusted for InsAUC_0–30_/GluAUC_0–30_**			**2hPG, adjusted for InsAUC_0–30_/GluAUC_0–30_ and Matsuda ISI**		
	**r**		***P***	**r**		***P***	**r**	***P***		**r**	***P***	
***Synthesis markers***												
Squalene	−0.008		0.834	−0.081		0.029	0.001	0.981		−0.059	0.110	
Lathosterol	0.097		0.008	0.0004		0.992	0.134	**2.9E-04**		0.034	0.359	
Cholestenol	0.091		0.013	0.008		0.826	0.118	**0.001**		0.041	0.273	
Desmosterol	0.203		**2.3E-08**	0.046		0.212	0.245	**2.0E-11**		0.065	0.080	
***Absorption markers***												
Cholestanol	−0.062		0.093	0.042		0.260	−0.098	0.008		0.004	0.914	
Campesterol	−0.131		**3.5E-04**	−0.030		0.417	−0.152	**3.6E-05**		0.048	0.195	
Sitosterol	−0.088		0.016	0.002		0.956	−0.110	**0.003**		0.072	0.053	
Avenasterol	−0.059		0.107	−0.007		0.848	−0.070	0.058		0.069	0.062	

Individuals with previously diagnosed diabetes and on statin or ezetimibe treatment were excluded (N = 727–740). Bold types indicate statistical significance with *P*≤0.006.

### Effects of Sterol Levels on the Worsening of Hyperglycemia and the Development of Incident Type 2 Diabetes

We first evaluated in a 5-year follow-up of the METSIM Study the effects of baseline sterol levels as predictors of the worsening of hyperglycemia, defined by a glucose area under the curve (Glucose AUC) in an OGTT (measurement of glucose levels at 0, 30 and 120 min) in 474 individuals without type 2 diabetes at baseline (Table 3). The levels of synthesis markers, cholestenol (*P* = 6.5×10^−4^) and desmosterol (*P* = 1.6×10^−4^) significantly predicted (*P*≤0.006) an increase in Glucose AUC after the adjustment for age, BMI, smoking and physical activity. In contrast, absorption markers campesterol, sitosterol and avenasterol significantly predicted a decrease in Glucose AUC at follow-up, after the adjustment for age, BMI, smoking and physical activity. The associations were largely attributable to low insulin sensitivity since additional adjustment for Matsuda ISI weakened all associations (Table 3), and abolished the association of desmosterol and campesterol with Glucose AUC at follow-up. Additional adjustment for insulin secretion, glucose AUC at baseline and total triglycerides did not substantially alter the results.

**Table 3:** Association between baseline sterol levels and glucose area under the curve (AUC) at the 5-year follow-up study.

Sterol, adjusted for total cholesterol	B (SE)	*P*	P*	*P^†^*	*P^‡^*
***Synthesis markers***					
Squalene	15.8 (54.1)	0.871	0.723	0.124	0.803
Lathosterol	142.8 (47.0)	**0.002**	0.140	0.879	0.259
Cholestenol	240.0 (56.0)	**1.1E-05**	**6.5E-04**	0.041	**0.003**
Desmosterol	376.7 (80.0)	**4.2E-07**	**1.6E-04**	0.256	0.193
***Absorption markers***					
Cholestanol	−280.9 (92.1)	**0.002**	0.065	0.617	0.051
Campesterol	−209.3 (42.2)	**1.7E-07**	**0.003**	0.151	0.043
Sitosterol	−214.0 (41.0)	**6.6E-08**	**0.002**	0.049	0.037
Avenasterol	−322.8 (64.6)	**1.7E-07**	**0.006**	0.038	0.052

Linear regression analysis, adjusted for age, BMI, smoking, physical activity and additional covariates as listed below. Individuals with previously diagnosed diabetes or with statin or ezetimibe treatment were excluded (N = 458–474). B, unstandardized beta, SE, standard error. Bold types indicate statistical significance with *P*≤0.006.

P, unadjusted.

P*, adjusted for age, BMI, smoking, and physical activity.

P^†^, adjusted for age, BMI, smoking, physical activity and Matsuda ISI.

P^‡^, adjusted for age, BMI, smoking, physical activity, Matsuda ISI, InsAUC_0–30_/GluAUC_0–30_, glucose AUC at baseline and total triglycerides.

A cholesterol synthesis marker desmosterol significantly predicted an increase, and absorption markers campesterol and avenasterol predicted a decrease in the risk of incident type 2 diabetes (Table 4). Associations of desmosterol, campesterol and avenasterol with incident type 2 diabetes were abolished after the adjustment for age, BMI, smoking and physical activity.

**Table 4:** Association between baseline sterol levels and newly developed type 2 diabetes in a 5-year follow-up study.

Sterol, adjusted for total cholesterol	Hazard ratio (95% CI)	*P*	P*	*P^†^*	*P^‡^*
*Synthesis markers*					
Squalene	1.48 (0.86–2.54)	0.153	0.253	0.368	0.446
Lathosterol	1.07 (1.01–1.13)	0.014	0.488	0.993	0.419
Cholestenol	1.45 (0.92–2.27)	0.106	0.845	0.610	0.707
Desmosterol	1.19 (1.05–1.35)	**0.005**	0.167	0.900	0.480
*Absorption markers*					
Cholestanol	0.89 (0.77–1.02)	0.097	0.327	0.849	0.974
Campesterol	0.95 (0.92–0.98)	**0.004**	0.084	0.434	0.934
Sitosterol	0.90 (0.83–0.97)	0.008	0.090	0.253	0.548
Avenasterol	0.60 (0.42–0.87)	**0.006**	0.158	0.789	0.607

Cox regression analysis. Participants with newly diagnosed type 2 diabetes at follow-up (N = 33) were compared to those with NGT/NFG and HbA1c <6.5% at baseline and follow-up examinations (N = 97). Diabetes diagnosis was defined as FPG ≥7.0mmol/l, 2hPG ≥11.1 mmol/l and/or HbA1c ≥6.5%. Individuals with previously diagnosed diabetes or with statin or ezetimibe treatment were excluded. Sterol levels are adjusted for total cholesterol and divided by 10. Bold types indicate statistical significance with *P*≤0.006.

P, unadjusted.

P* adjusted for age, BMI, smoking, and physical activity.

P^†^ adjusted for age, BMI, smoking, and physical activity and Matsuda ISI.

P^‡^ adjusted for age, BMI, smoking, and physical activity and Matsuda ISI, InsulinAUC_0–30_/GlucoseAUC_0–30_, glucose AUC at baseline, and total triglycerides.

### SNPs Regulating Sterol Levels

**Figure 2:**
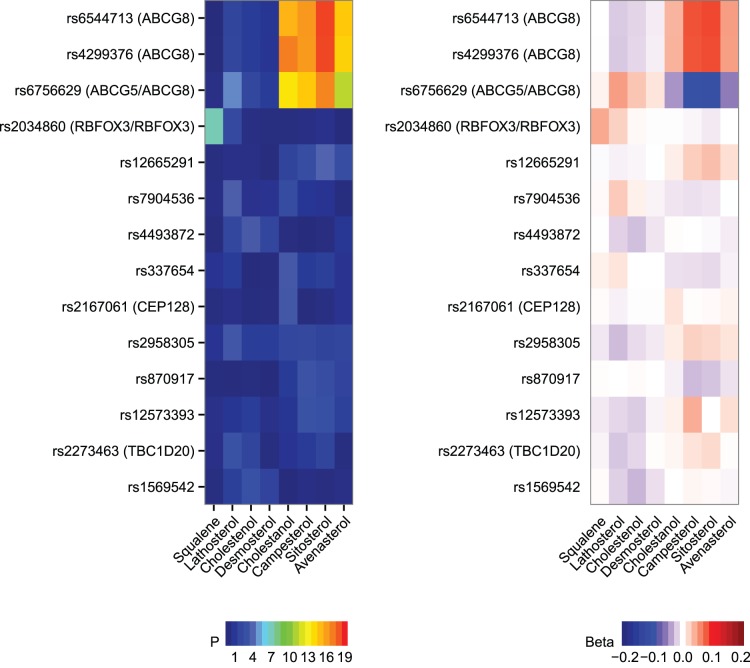
SNPs associated with sterol levels (left panel) and the effect size evaluated by the standardized β coefficient from linear regression model (right panel). Genotyping results represent a meta-analysis of 1^st^ stage (GWAS, EUGENE2 Study) and 2^nd^ stage of targeted replication (METSIM Study), total N = 1,013. SNPs having an association *P*<0.001 are shown. Individuals with previously diagnosed diabetes and on statin or ezetimibe treatment were excluded. *P*-values are adjusted for age, gender and BMI.

We performed a genome-wide association study of 538,265 SNPs and non-cholesterol sterol levels in the Kuopio cohort of the EUGENE2 study, and a targeted replication of 134 SNPs in the METSIM cohort. Table 5 presents genome-wide significant and Figure 2 nominally significant results (*P*<0.001) of a meta-analysis of the discovery sample and the replication sample. The three genome-wide significant SNPs were all associated with the cholesterol absorption marker levels, rs4299376 (from *P* = 4.7×10^−15^ to *P* = 4.3×10^−19^) and rs6544713 (*P* = 2.5×10^−15^ to 3.7×10^−19^, linkage disequilibrium in European populations with rs429976, r^2^ = 0.99) of *ABCG8*, and rs6756629 of *ABCG5/ABCG8* (from *P* = 4.8×10^−12^ to *P* = 1.8×10^−17^). Effect sizes, calculated as per minor allele, were positive for the *ABCG8* SNPs and negative for rs6756629 of *ABCG5/ABCG8*. Genome-wide significant SNPs (rs4299376, rs6544713, and rs6756629) were all associated with the cholesterol absorption marker levels even after the exclusion of participants with previously diagnosed diabetes and those taking statin medication, and adjustment for age, gender and BMI (Table 5). The major (sterol-decreasing) allele of the most significant SNP, rs4299376, was associated with elevated levels of FPG (*P* = 0.022) in the additive model, but not with the 2hPG (*P* = 0.425) or Glucose AUC (*P* = 0.144) in the cross-sectional METSIM Study cohort (5,639 men without previously diagnosed diabetes and without statin treatment). *ABCG8* rs4299376 did not predict the worsening of hyperglycemia in a 5-year follow-up study (P≥0.121 for association with FPG, 2hPG and Glucose AUC at follow-up in the additive model).

**Table 5:** Genotyping results: 1^st^ stage results obtained from genome-wide association study (EUGENE2 Study), 2^nd^ stage results obtained from replication study (METSIM Study), and meta-analysis of both cohorts.

						1^st^ stage (EUGENE2)			2^nd^ stage (METSIM)			Meta-analysis	
		Ref. allele	Other allele	Ref. allele frequency(1st stage)	Ref. allele frequency(2nd stage)	N	β	*P*	N	β	*P*	B	*P*
***Cholestanol***													
*ABCG8*	rs4299376	C	A	0.19	0.15	271	0.037	5.6E-05	734	0.039	1.2E-13	0.038	1.1E-17
*ABCG8*	rs6544713	A	G	0.19	0.15	267	0.036	1.7E-04	738	0.038	1.8E-12	0.037	9.9E-16
*ABCG5/8*	rs6756629	A	G	0.10	0.10	268	−0.053	7.4E-06	738	-0.043	8.2E-10	−0.046	2.2E-14
***Campesterol***													
*ABCG8*	rs6544713	A	G	0.19	0.15	267	0.108	3.5E-08	738	0.075	2.1E-10	0.084	6.4E-17
*ABCG8*	rs4299376	C	A	0.19	0.15	271	0.105	3.9E-08	734	0.075	2.5E-10	0.083	7.3E-17
*ABCG5/8*	rs6756629	A	G	0.10	0.10	268	−0.136	1.6E-08	738	−0.089	1.1E-08	−0.102	2.5E-15
***Sitosterol***													
*ABCG8*	rs6544713	A	G	0.19	0.15	267	0.104	1.8E-08	738	0.081	4.6E-12	0.088	3.7E-19
*ABCG8*	rs4299376	C	A	0.19	0.15	271	0.101	1.9E-08	734	0.081	5.6E-12	0.087	4.3E-19
*ABCG5/8*	rs6756629	A	G	0.10	0.10	268	−0.139	1.0E-09	738	−0.094	1.3E-09	−0.108	1.8E-17
***Avenasterol***													
*ABCG8*	rs6544713	A	G	0.19	0.15	267	0.055	2.5E-05	738	0.048	3.7E-11	0.050	2.5E-15
*ABCG8*	rs4299376	C	A	0.19	0.15	271	0.054	2.5E-05	734	0.047	7.0E-11	0.049	4.7E-15
*ABCG5/8*	rs6756629	A	G	0.10	0.10	268	−0.090	1.8E-08	738	−0.045	3.1E-06	−0.057	4.8E-12

Only results from meta-analysis indicating a genome-wide significance (P<5×10^−8^) are shown. β = Regression coefficient. Individuals with previously diagnosed diabetes and on statin or ezetimibe treatment are excluded. P values adjusted for age, gender, and BMI.

## Discussion

We demonstrated for the first time that non-cholesterol sterol levels predicted the worsening of hyperglycemia and the development of incident type 2 diabetes in a 5-year prospective follow-up of a Finnish population-based study including 1,050 men (the METSIM cohort). We also demonstrated that rs4299376 and rs6544713 of *ABCG8* and rs6756629 of *ABCG5/8* were significantly associated with all cholesterol absorption markers (*P* for association from 4.8×10^−12^ to 3.7×10^−19^), but not with cholesterol synthesis markers. The major allele of the most significant SNP, rs4299376 of *ABCG8*, was nominally associated with elevated levels of FPG at baseline, but did not predict the development of hyperglycemia in a 5-year follow-up study.

Elevated cholesterol synthesis and reduced absorption have been reported in individuals with type 2 diabetes or hyperglycemia 3–6,8–9, and have been associated with insulin resistance [7] in cross-sectional studies. However, none of the previous studies has investigated the association of sterol levels in the entire range of hyperglycemia. We showed that in a cross-sectional setting, cholesterol synthesis markers were elevated and cholesterol absorption markers decreased as a function of 2hPG and FPG levels, respectively.

In our 5-year follow-up of the METSIM cohort we found that the cholesterol synthesis markers cholestenol and desmosterol significantly (P≤0.006) predicted increases in Glucose AUC, and desmosterol also incident type 2 diabetes. Adjustment for Matsuda ISI alone largely abolished these associations demonstrating that low insulin sensitivity was the major mechanism explaining the worsening of hyperglycemia and the development of incident diabetes. Absorption markers campesterol, sitosterol, and avenasterol significantly predicted a decrease in Glucose AUC, and campesterol and avenasterol also a lower risk of incident type 2 diabetes. Statistically significant associations were abolished when adjusted for Matsuda ISI and other confounding factors. To the best of our knowledge, this is the first prospective study demonstrating that non-cholesterol sterols predict the worsening of hyperglycemia and incident type 2 diabetes.

We also investigated the role of genetic factors in the association of sterol levels with the risk of type 2 diabetes. We showed that rs4299376 and rs6544713 (in high linkage disequilibrium with rs4299376) of *ABCG8* and rs6756629 of *ABCG5/8* were very significantly associated with plant sterols (campesterol, sitosterol and avenasterol) and cholestanol (β from 0.038 to 0.087, *P*<1×10^−14^ for rs4299376, and β from −0.046 to −0.108, *P*<1×10^−11^ for rs6756629) after adjustment for age, gender and BMI. The ATP hemitransporter proteins ABCG5 and ABCG8 are key regulators of dietary cholesterol absorption in the intestinal lumen. SNP rs4299376 of *ABCG8* has previously been associated with LDL cholesterol level and rs6756629 of *ABCG5/8* with total cholesterol level [25], [26]. Previously published GWAS identified two SNPs of *ABCG8* (rs41360247 and rs4245791) as regulators of plant sterol (campesterol and sitosterol) levels [14]. Our results are in agreement with these findings as SNPs identified in our study are in close linkage equilibrium (r^2^ = 0.99–1.0) with the SNPs reported earlier [14]. However, the major allele of rs4299376 of *ABCG8,* which was very significantly associated with surrogate markers of cholesterol absorption, was nominally associated with elevated fasting glucose in 5,639 men in cross-sectional analysis of the METSIM cohort (*P* = 0.022), but not with the worsening of hyperglycemia or the development of incident type 2 diabetes in our follow-up study, probably due to a relatively small sample size in our follow-up study.

Our study has limitations. Only Finnish men were included in the METSIM Study, and therefore, we do not know whether our results are applicable to women and to different ethnic or racial groups. We had only modest power to demonstrate statistically significant associations of gene variants with non-cholesterol sterol levels.

In conclusion, our population-based study is the first to show that desmosterol, marker of cholesterol synthesis, and campesterol and avenasterol, markers of cholesterol absorption, significantly predict the worsening of hyperglycemia and incident type 2 diabetes. The predominant mechanism linking sterol metabolism to hyperglycemia is likely to be low insulin sensitivity.

## Supporting Information

Table S1 Mean level and the range of sterol levels at baseline (N = 746).
(DOCX)
