# Supplementary material for: Non-Cholesterol Sterol Levels Predict Hyperglycemia and Conversion to Type 2 Diabetes in Finnish Men
Source: PLoS One. 2013 Jun 28;8(6):e67406. doi: 10.1371/journal.pone.0067406 (PMC3696087; doi:10.1371/journal.pone.0067406)
Supplement: Table S1 — Mean level and the range of sterol levels at baseline (N = 746). (DOCX) [file pone.0067406.s001.docx]

**TABLE S1.** Mean level and the range of sterol levels at baseline (N=746)

| **Sterol, adjusted for total cholesterol**  **(10^2^ mmol/mol of cholesterol)** | **Mean** | **SD** | **Range** |
| --- | --- | --- | --- |
| Squalene | 19.0 | 9.9 | 7.9 - 190.3 |
| Lathosterol | 148.7 | 56.6 | 23.5 - 408.6 |
| Cholestenol | 23.4 | 13.6 | 6.4 - 306.2 |
| Desmosterol | 94.1 | 35.3 | 48.4 - 698.7 |
| Cholestanol | 142.4 | 29.4 | 45.3 - 399.4 |
| Campesterol | 264.2 | 119.7 | 52.0 - 913.7 |
| Sitosterol | 129.0 | 66.9 | 37.4 - 540.4 |
| Avenasterol | 41.6 | 12.6 | 14.7 - 120.9 |

Individuals with previously diagnosed diabetes or with statin or ezetimibe treatment were excluded.
